# Robotic versus laparoscopic surgery for rectal cancer in male urogenital function preservation, a meta-analysis

**DOI:** 10.1186/s12957-018-1499-y

**Published:** 2018-10-02

**Authors:** Xiaoli Tang, Zheng Wang, Xiaoqing Wu, Meiyuan Yang, Daorong Wang

**Affiliations:** 1 Department of General Surgery, The Second Xiangya Hospital of Central South University, Renmin Road No.139, Changsha, 410001 China; 2 Department of General Surgery, Medical College of Yangzhou University, Huaihai Road No.7, Yangzhou, 225001 China; 3 Department of General Surgery, The northern Jiangsu people’s Hospital, Nantong Road No.98, Yangzhou, 225001 China

## Abstract

**Background:**

Urogenital dysfunction after rectal cancer surgery can largely affect patients’ postoperative quality of life. Whether robotic surgery can be a better option when comparing with laparoscopic surgery is still not well-known.

**Methods:**

Comprehensive search in PubMed, Embase, Cochrane Library, and Clinical Trials was conducted to identify relevant studies in March 2018. Studies comparing robotic surgery with laparoscopic surgery were included. Measurement of urogenital function was through the International Prostate Symptom Score and International Index of Erectile Function.

**Results:**

Six studies with 386 patients in robotic group and 421 patients in laparoscopic group were finally included. Pooled analysis indicated that bladder function was better at 12 months in the robotic group after the procedures (mean difference, − 0.30, 95% CI, − 0.52 to − 0.08). No significant difference was found at 3 and 6 months postoperatively (mean difference, − 0.37, 95% CI, − 1.48 to 0.73; mean difference, − 1.21, 95% CI, − 2.69 to 0.28). Sexual function was better at 3 months in the robotic group after surgery (mean difference, − 3.28, 95% CI, − 6.08 to − 0.49) and not significantly different at 6 and 12 months. (mean difference, 3.78, 95% CI, − 7.37 to 14.93; mean difference, − 2.82, 95% CI, − 8.43 to 2.80).

**Conclusion:**

Robotic surgery may offer faster recovery in urogenital function compared to laparoscopic surgery for rectal cancer.

## Background

Rectal cancer is one of the most common malignant neoplasm worldwide [1, 2]. Great improvement in management of rectal cancer has been made over the past few decades, such as recommendation for early screening in high-risk population and use of adjuvant and neoadjuvant chemotherapy [3–5]. However, even with lots of newly invented treatments, surgery is still the only curative treatment for rectal cancer to achieve radical resection so the patient can gain oncological safety. In the past two decades, minimal invasive surgery like laparoscopy has been accepted worldwide. Existed randomized control trials have proved the certain superiority of laparoscopy over conventional open surgery with equal oncological safety [6–8]. Robotic surgery was first used in colorectal disease in 2001 [9], since then, it has gained great popularity around the world as it overcomes some technical limitations compared to laparoscopic surgery. Although the main goals of rectal surgery are accomplishing adequate distal and circumferential margins, postoperative function outcomes like sexual and urological functions greatly influence postoperative psychological well-being and account for a large part of patients’ quality of life [10–13]. Previous studies have illustrated urogenital impairment after rectal surgery with approximately 5% of patients suffer permanent bladder dysfunction or impotence problem [14, 15]. When compared to laparoscopy, whether robotic surgery can be a better option regarding recovery of sexual and urological function is still under great debate. The present study aimed at answering this question with current available evidence by conducting a meta-analysis.

## Methods

**Fig. 1:**
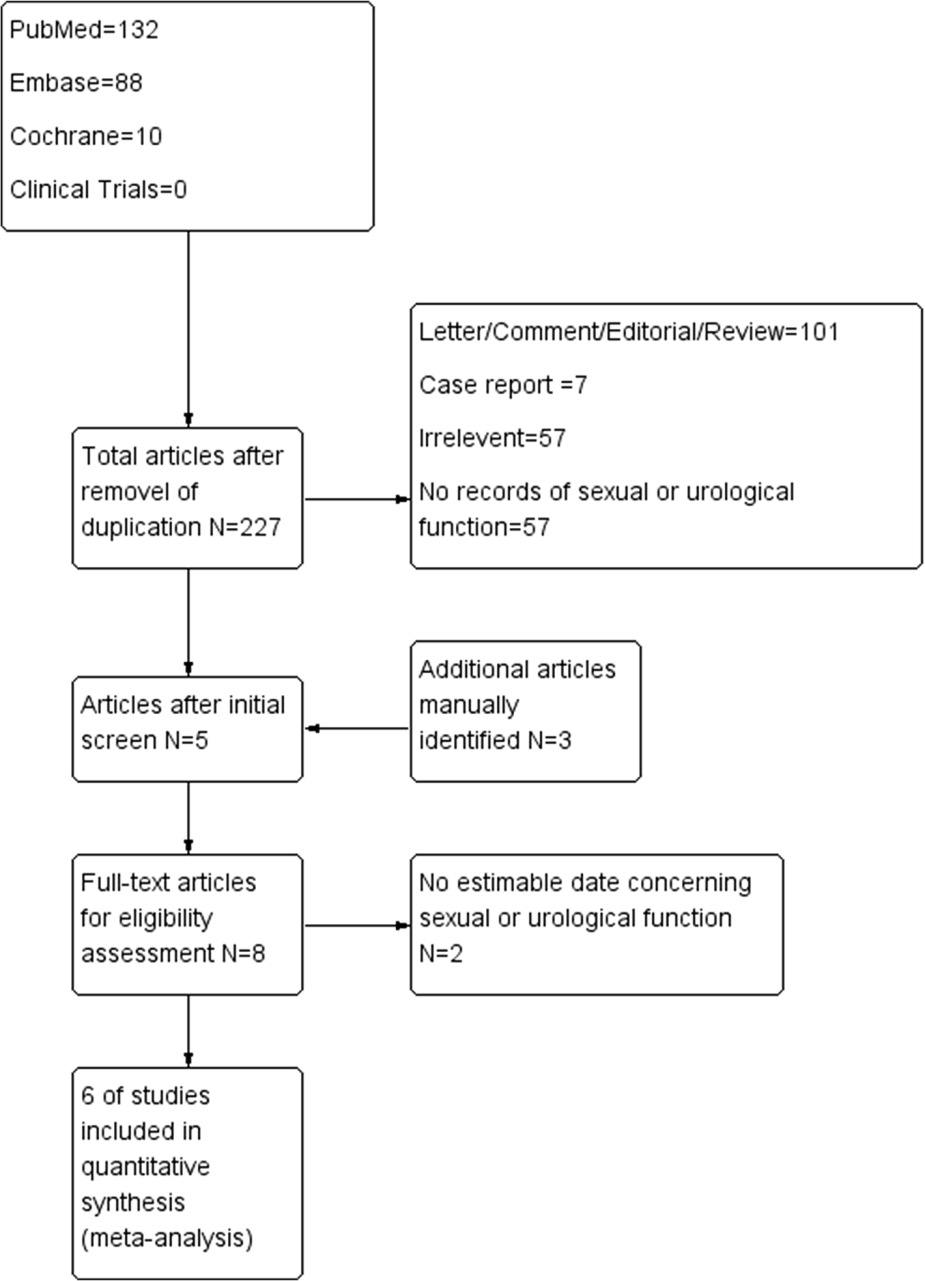
Study screening flow

A comprehensive search was conducted in March 2018 within PubMed, Embase, Cochrane Library, and Clinical Trials. The searching terms were “Colorectal Neoplasms” [Mesh] + “Laparoscopy” [Mesh] + “Robotic Surgical Procedures” [Mesh] + “sexual dysfunction” or “sexual impairment” + “urological dysfunction” or “urological impairment.” Clinical studies from January 2001 till the search day which compared robotic surgery with laparoscopic surgery with sexual or urological outcomes as primary or secondary endpoints were identified for further screening, as well as studies containing a subgroup of participants whose urogenital functions were recorded. We included studies both designed as randomized control trials or observational studies. Non-human papers, comment, letter, correspondence, review, expert opinions, and case reports were excluded. Studies with irrelevant topics and studies with no records regarding sexual and urological function were excluded as well. The screening process was shown in Fig. 1. Two researchers independently screened the articles without any consult. If any disagreement occurred, the article was brought into discussion to decide whether it will be included. Data extraction from each enrolled study mainly included author, year, study design, information feasible for quality evaluation, patients baseline date, tumor-related information, operative procedure, and functional outcomes both preoperatively and postoperatively. The Review Manager software (version 5.3) from Cochrane was used to analyze the extracted data under the instruction of Cochrane handbook.

## Results

After screening, six studies [16–21] were included in this meta-analysis. Three hundred and eighty-six patients in total underwent robotic surgery and 421 patients underwent laparoscopic surgery. Among six studies, four of them were retrospectively designed [16–19] and the other two were randomized control trials (RCT) [20, 21]. We used the Newcastle–Ottawa scale to evaluate the quality of observational studies (shown in Table 1) and the risks of bias system from Cochrane to assess the quality of RCTs. Basic characteristics of the studies were summarized in Table 2.

**Table 1:** NOS scale for observational studies

Study	Selection				Comparability	Outcome assessment			Score
	1	2	3	4	5, 6	7	8	9	
D’ Annibale 2013	*	*	*	*	*, *	*	*	*	9
Panteleimonitis 2016	*	*	*	*	*, 0	*	*	*	8
Park 2014	*	*	*	*	*, *	*	*	*	9
Kim 2012	*	*	*	*	*, *	*	*	*	9

Explanation

1: Adequate definition of the cases, study-enrolled cases with independent validation. (yes, *; no or not reported, 0)

2: Representative of the cases, consecutive or obviously representative cases. (yes, *; no or not reported, 0)

3: Selection of controls, community controls. (yes, *; no or not reported, 0)

4: Clear definition of the controls, no previous history of the same procedure. (yes, *; no or not reported, 0)

5: Comparability of cases and controls on the basis of the design or analysis, the patients baseline characteristics were similar between different groups. (yes, *; no or not reported, 0)

6: Comparability of cases and controls for other factors, the same type of procedure, the same surgical team to perform the procedure. (yes, *; no or not reported, 0)

7: Ascertainment of exposure, complete surgical records. (yes, *; no or not reported, 0)

8: Same method of ascertainment for cases and controls. (yes, *; no or not reported, 0)

9: Adequacy of follow up of cohorts (yes, *; no or not reported, 0)

**Table 2:** Characteristics of the included studies

Author	Year	Country	Study design	No. of robotic procedures	No. of laparoscopic procedures	Methods of function assessment
Wang	2016	China	RCT	71	66	IPSS, IEFF
Jayne	2017	UK	RCT	175	176	IPSS, IEFF
Panteleimonitis	2016	UK	Retrospective	48	78	IPSS,IEFF
Park	2014	Korea	Retrospective	32	32	IPSS, IEFF
Kim	2012	Korea	Retrospective	30	39	IPSS, IEFF
D‘Annibale	2013	Italy	Retrospective	30	30	IPSS, IEFF

Abbreviation: *UK*, United Kingdom; *RCT*, randomized controlled trial; *IPSS*, International Prostate Symptom Score; *IEFF*, International Index of Erectile Function

### Urological function

**Fig. 2:**
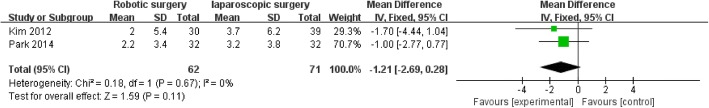
IPSS change from baseline at 3 months postoperatively

**Fig. 3:**
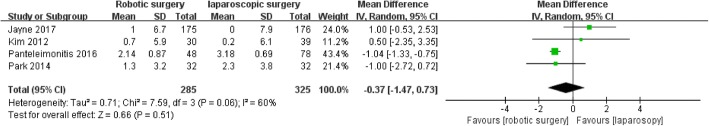
IPSS change from baseline at 6 months postoperatively

**Fig. 4:**
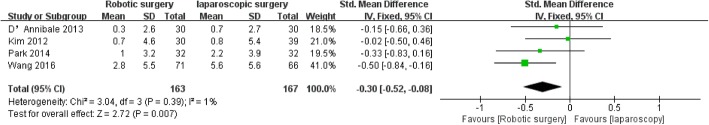
IPSS change from baseline at 12 months postoperatively

**Fig. 5:**
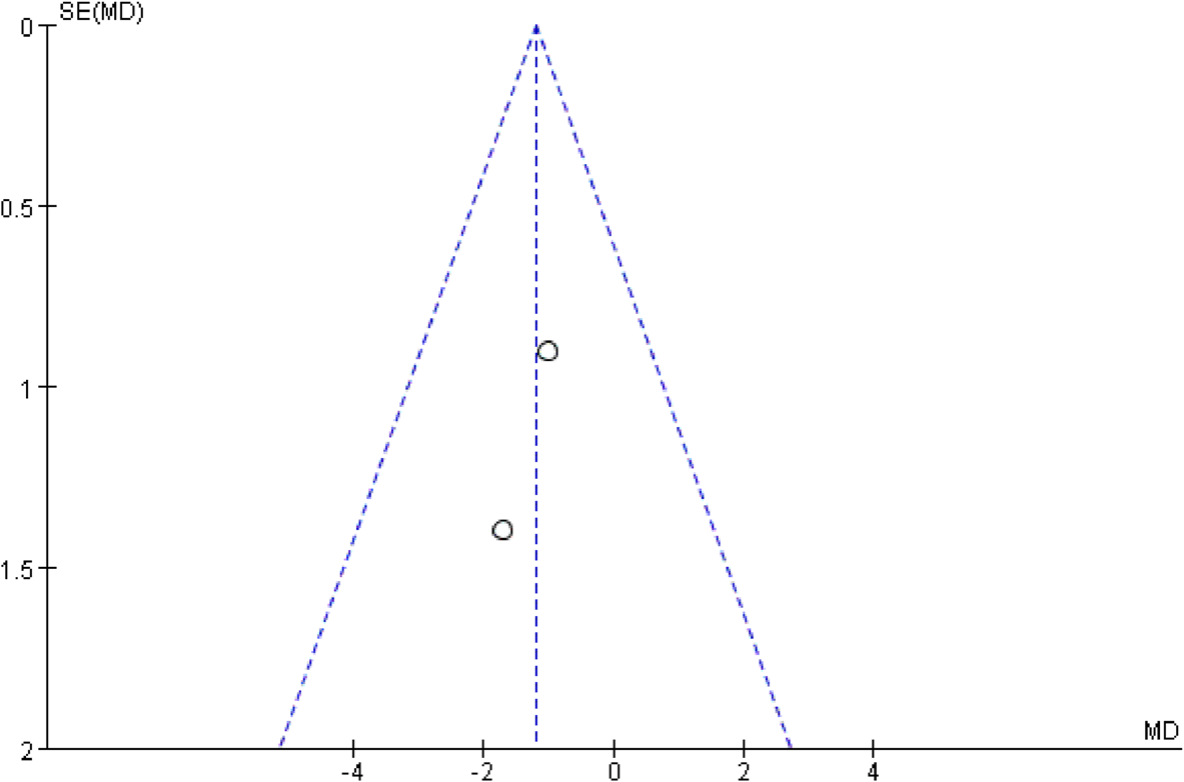
Funnel plot for IPSS at 3 months

**Fig. 6:**
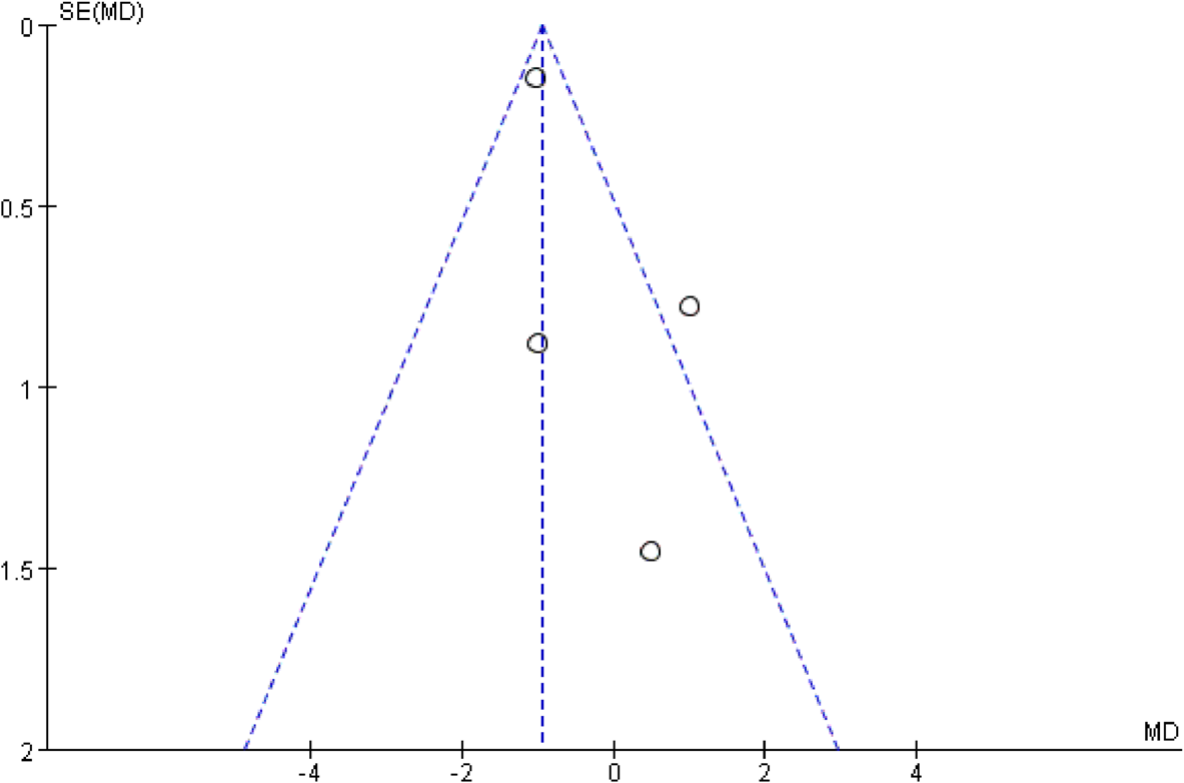
Funnel plot for IPSS at 6 months

**Fig. 7:**
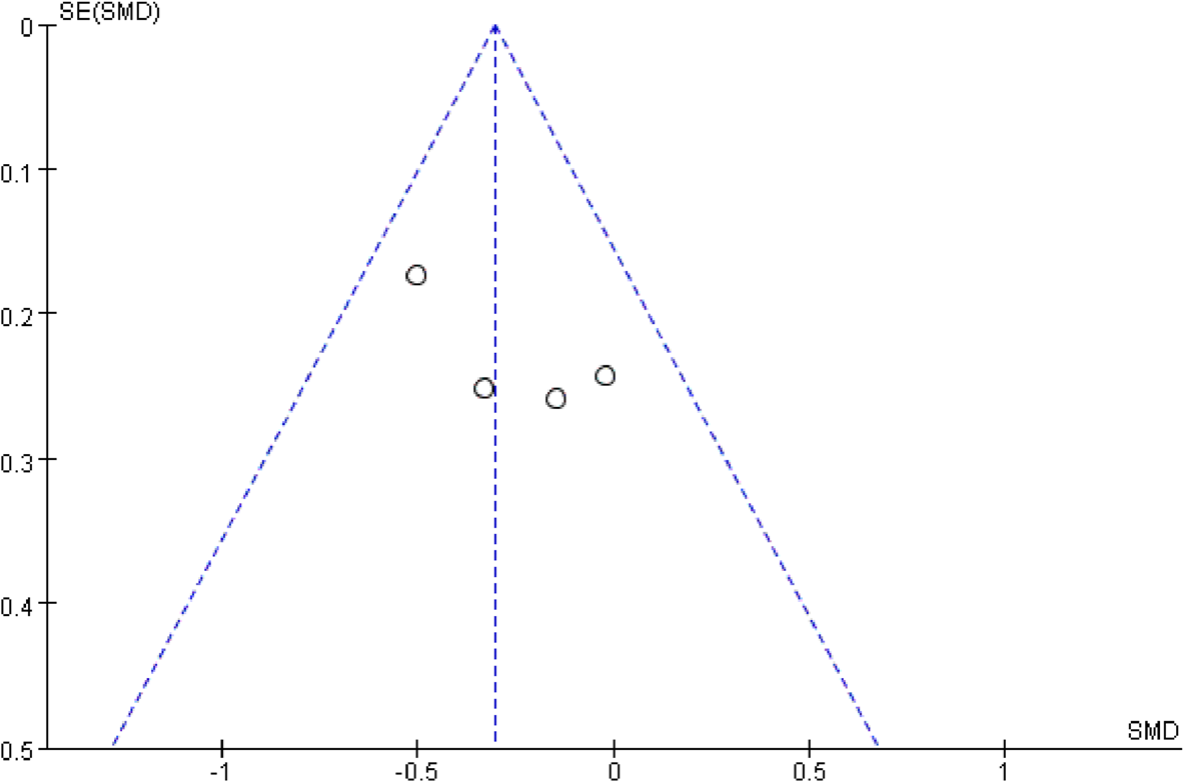
Funnel plot for IPSS at 12 months

All studies used the International Prostate Symptom Score (IPSS) to evaluate the patients urological function mainly concerning seven aspects as bladder emptying, frequency, intermittency, nocturia, urgency, straining, and weak stream. Each aspect of the scale ranges from 0 to 6 points with higher scores indicate worse function. All studies recorded IPSS preoperatively as baseline status. To minimize heterogeneity among different religions regarding sexual and urological functions, we used the change in the scores from baseline to analyze the difference. Two studies reported IPSSs 3 months after surgery. The pooled estimate indicated that there was no significant difference between the two groups. (mean difference, − 1.21, 95% CI, − 2.69 to 28, *p* = 0.11). No heterogeneity was found among studies. Four studies recorded IPSSs 6 months after the surgery, and the result showed no significant difference between laparoscopy and robotic procedure (mean difference, − 0.37 95% CI − 1.47 to 0.73, *p* = 0.51). Moderate heterogeneity was found among studies with *I*^2^ = 60%, so the random effect model was used and publication bias was detected by conducting the funnel plot (Fig. 5). Four studies reported IPSSs of 12 months after the surgery, and the result favored robotic surgery (mean difference, − 0.30 95% CI, − 0.52 to − 0.08 *p* = 0.007). Almost no heterogeneity was found among studies with *I*^2^ = 1%. Forest plots and funnel plots were shown in Figs. 2, 3, 4, 5, 6, and 7.

### Sexual function

**Fig. 8:**
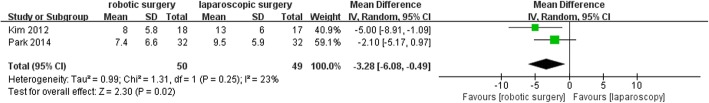
IIEF score change from baseline at 3 months postoperatively

**Fig. 9:**
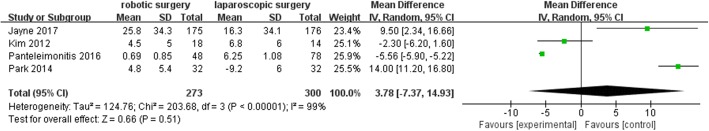
IIEF score change from baseline at 6 months postoperatively

**Fig. 10:**
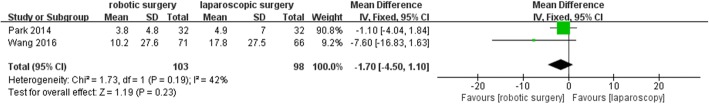
IIEF score change from baseline at 12 months postoperatively

**Fig. 11:**
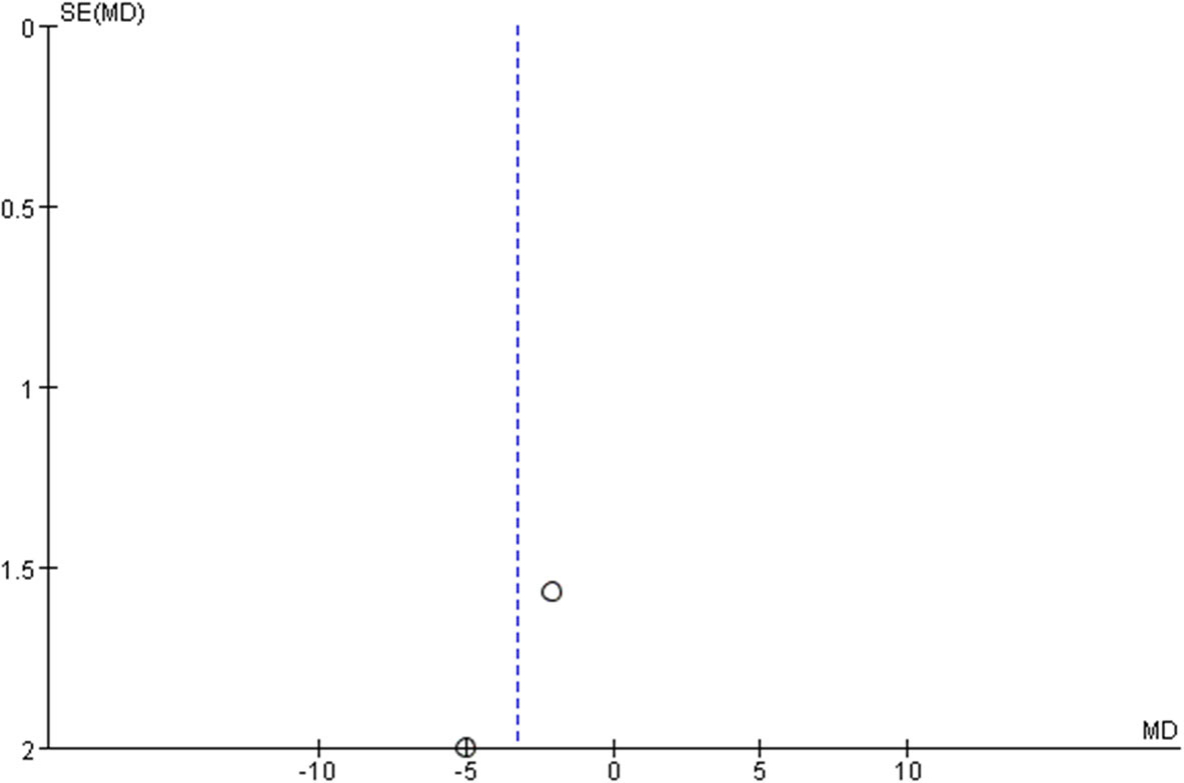
Funnel Plot for IIEF at 3 months

**Fig. 12:**
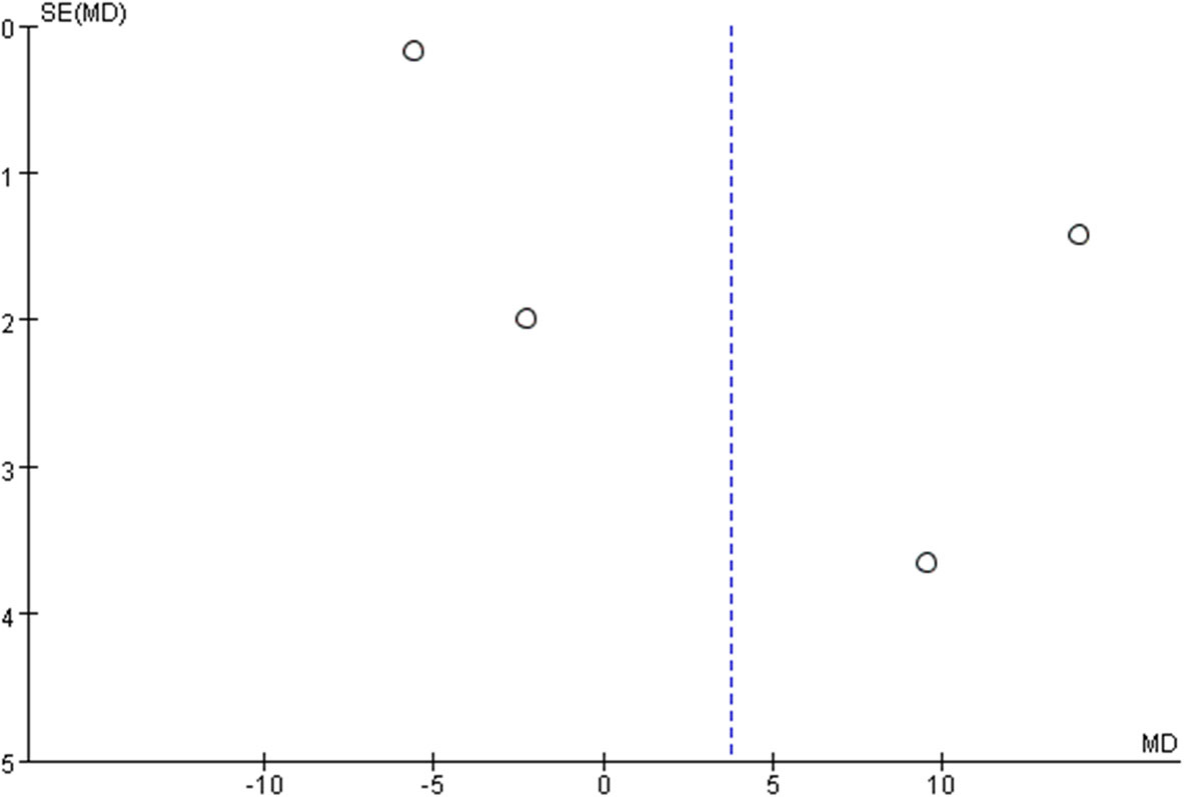
Funnel Plot for IIEF at 6 months

**Fig. 13:**
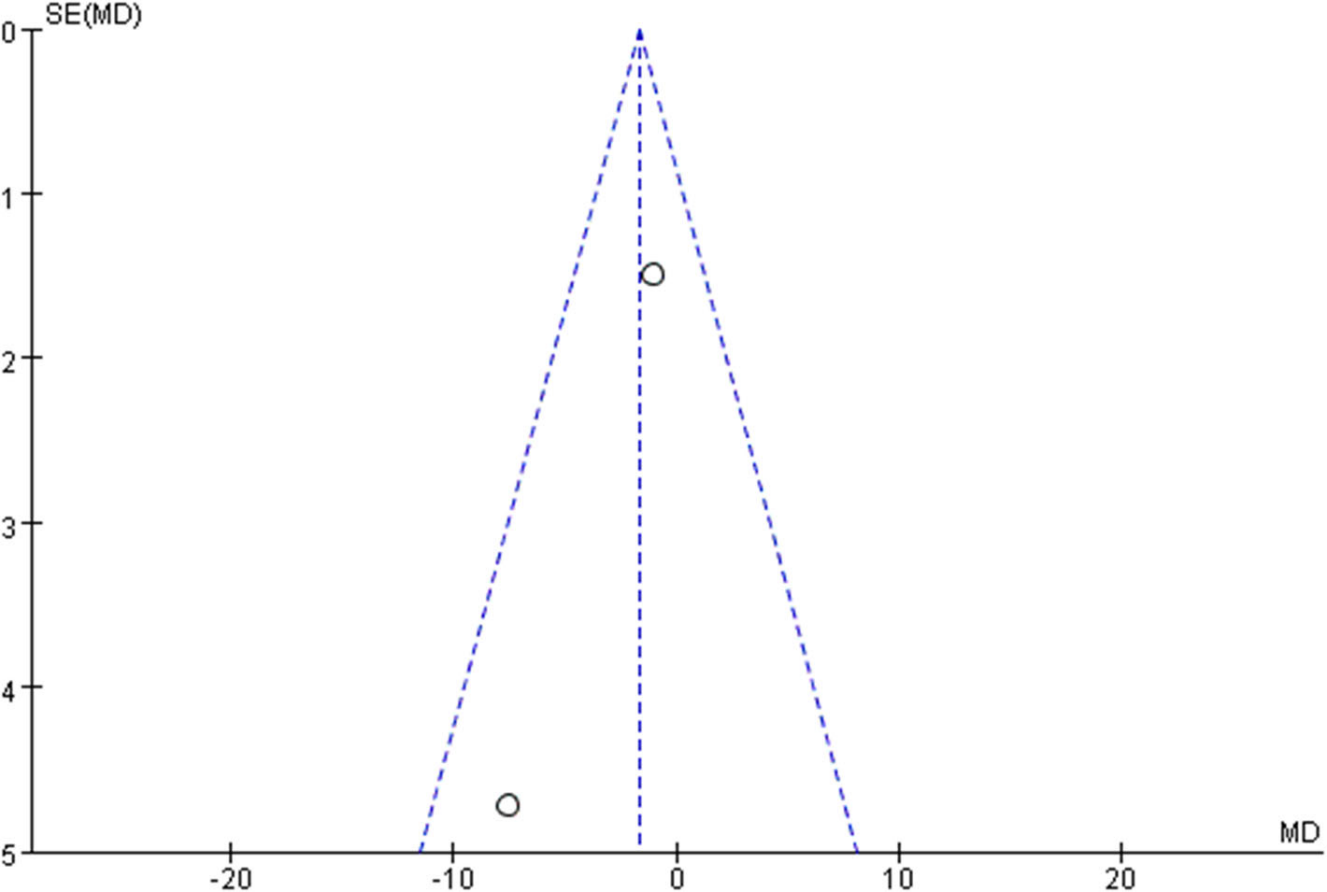
Funnel Plot for IIEF at 12 months

All studies used the International Index of Erectile Function (IIEF) score to assess patients’ sexual function. The IIEF is a well-recognized self-report questionnaire scale which contains five factors as erectile function, orgasmic function, libido, intercourse satisfaction, and overall satisfaction [22]. The higher scores also indicated better sexual function. To minimize the impact of heterogeneity among different studies, we used the change from baseline date of each study to analyze. Only two studies reported IIEF at 3 months after surgery, and the result favored robotic surgery (mean difference − 3.28, 95% CI − 6.08 to − 0.49, *p* = 0.02). Four studies recorded IIEF scores at 6 months after surgery, and the result showed no significant difference between the two groups (mean difference, 3.78 95% CI − 7.37 to 14.93, *p* = 0.51). Great heterogeneity was found among studies with *I*^2^ = 99%. Two studies reported IIEF scores at 12 months after surgery, and the result showed no significant difference among the two groups (mean difference, − 2.82, 95% CI, − 8.43 to 2.80). Moderate heterogeneity was found with *I*^2^ = 42%. The forest plots and funnel plots of IIEF were shown in Figs. 8, 9, 10, 11, 12 and 13.

## Discussion

Robotic surgery for colorectal cancer has been widely accepted over the past decade. High-quality evidence such as RCTs and meta-analysis has suggested that robotic surgery can achieve oncological safety compared to laparoscopy with lower conversion rate and faster recovery [23, 24]. However, it is still not well explored whether the advantages of robotic surgery can translate into better urogenital function after the procedure. Few previously published meta-analyses have tried to answer this question with available evidence. For the specific topic of urogenital function outcomes, Malene Broholm et al. conducted a meta-analysis with 10 studies enrolled [25]. They suggested that IPSS was better at 3 months and 12 months after surgery in robotic surgery group. As for IIEF score, they found better results in robotic group at both 3 and 6 months after surgery. However, they found that the feasible data from these 10 studies were scarce; thus the results should be interpreted cautiously. Another meta-analysis conducted by Lee et al. found that robotic patients had a better IPSS at 3 months after surgery, but this superiority did not present at 6 months and 12 months [23]. As for sexual function, researchers found that patients in robotic surgery had better IIEF scores at both 3 and 6 months postoperatively. However, they also claimed limitations in their study, like limited data and vague information about follow-ups. They were also concerned about the impact of equipment learning curve on postoperative outcomes because all the procedures were not performed by the same surgical team. Panteleimonitis et al. did a critical analysis of currently available evidence of urogenital function following robotic surgery for rectal cancer [26]. They searched the literature for studies of robotic surgery without conducting a meta-analysis due to great heterogeneity. They concluded that there seemed to be a trend towards better urogenital function following robotic surgery when comparing with laparoscopic surgery. However, they found that many identified studies were not well-designed, so that it was not feasible to form a high-quality evidence based on the situation.

The present study found that IPSSs at 12 months were better after robotic surgery. No significant difference was found between laparoscopic and robotic procedures at 3 and 6 months. However, previous studies have indicated that the minimum perceptible differences detected by IPSS should be more than 3 points [27]. Our result showed that the pooled difference between the two groups was only 0.3. Therefore, this significant difference should be interpreted cautiously. Further evidence with larger samples and more comprehensive investigation of urological function is needed to form a more solid conclusion. As for sexual function recovery, the study found that at 3 months after the procedure, patients that underwent robotic surgery scored better at IIEF. This difference was not found at 6 months and 12 months.

Normal bladder and sexual function were regulated by intact supply of parasympathetic and sympathetic nerve. These regulation nerves usually lie among the pelvic side-walls which make them susceptible to be injured during rectal resection [28]. Although the appearance of urogenital dysfunction is polyfactorial, iatrogenic damage during surgery is thought to be the main cause [29–31]. In addition, urogenital dysfunction after the procedures largely depends on perioperative damage to the autonomic nerve and the site of anastomosis [11, 32]. In conventional laparoscopic surgery, the leading surgeon had to dissect the rectum in a narrow pelvic space with stiff equipment. In these cases, the autonomic nerve lying among the pelvic walls are easily damaged especially when the tumor is bulky [33]. Robotic surgery is supposed to conquer these technical limitations due to its flexible-wristed tremor-free instruments which mimic the surgeon’s hands. In addition, based on a stable platform, the camera, which can provide a high-definition 3D image, is easier to control. These advantages should theoretically benefit patients with better nerve preservation, thus better postoperative functional outcomes.

The present meta-analysis has certain limitations. The most important one is that many detail information concerning the height of anastomosis and type of surgery, whether the patients were sexually active before the procedures, are not mentioned in the original studies. We figured that it is one of the reasons for great heterogeneity among studies. In addition, lack of detailed information can also bring great confounding factors which made the result less reliable. Another limitation is scarce data. Although we included newly published studies, the estimable data for each result is still not abundant enough to establish a solid conclusion. However, we did find it crucial to provide necessary education and counseling about possible urogenital dysfunction after rectal surgery to help patients facilitate realistic expectation and psychological preparation, especially in preoperative sexually active patients [34, 35].

## Conclusion

Our study formed a primary result that rectal cancer patients underwent robotic surgery may recover faster in urological functions 12 months postoperatively. As for sexual function recovery, patients gained better sexual function at 3 months postoperatively in robotic group while no significant difference was found between robotic surgery and laparoscopic surgery at 6 and 12 months. Future well-designed, larger enrolled participant studies are needed to further address this question for rectal cancer patients.

## Abbreviations

CI: Confidence interval
IIEF: International Index of Erectile Function
IPSS: International Prostate Symptom Score
RCT: Randomized control trials
